# Multi-Label Classification of Chest X-ray Abnormalities Using Transfer Learning Techniques

**DOI:** 10.3390/jpm13101426

**Published:** 2023-09-22

**Authors:** Jakub Kufel, Michał Bielówka, Marcin Rojek, Adam Mitręga, Piotr Lewandowski, Maciej Cebula, Dariusz Krawczyk, Marta Bielówka, Dominika Kondoł, Katarzyna Bargieł-Łączek, Iga Paszkiewicz, Łukasz Czogalik, Dominika Kaczyńska, Aleksandra Wocław, Katarzyna Gruszczyńska, Zbigniew Nawrat

**Affiliations:** 1 Department of Biophysics, Faculty of Medical Sciences in Zabrze, Medical University of Silesia, Jordana 19, 41-808 Zabrze, Poland; dkrawczyk@frk.pl (D.K.); nawrat@frk.pl (Z.N.); 2 Department of Radiology and Nuclear Medicine, Faculty of Medical Sciences in Katowice, Medical University of Silesia, 40-752 Katowice, Poland; kgruszczynska@sum.edu.pl; 3 Professor Zbigniew Religa Student Scientific Association at the Department of Bio-Physic, Faculty of Medical Sciences in Zabrze, Medical University of Silesia, Jordana 19, 41-808 Zabrze, Poland; michalbielowka01@gmail.com (M.B.); marcinarojek@gmail.com (M.R.); adam.mitrega2306@gmail.com (A.M.); domika1313@gmail.com (D.K.); igapaszkiewicz.ip@gmail.com (I.P.); lukczog@gmail.com (Ł.C.); dominikakaczynska01@gmail.com (D.K.); aleksandrabwoclaw@gmail.com (A.W.); 4 Individual Specialist Medical Practice, 40-754 Katowice, Poland; maciejmichalcebula@gmail.com; 5 Psychiatry Ward, Provincial Specialist Hospital No. 4, 41-902 Bytom, Poland; mbielowka@op.pl; 6 Paediatric Radiology Students’ Scientific Association at the Division of Diagnostic Imaging, Department of Radiology and Nuclear Medicine, Faculty of Medical Science in Katowice, Medical University of Silesia, 40-752 Katowice, Poland; katarzyna.bargiel@op.pl; 7 Foundation of Cardiac Surgery Development, 41-800 Zabrze, Poland

**Keywords:** deep learning, machine learning, radiology, X-ray, diagnostic classification, CNN, chest X-ray

## Abstract

In recent years, deep neural networks have enabled countless innovations in the field of image classification. Encouraged by success in this field, researchers worldwide have demonstrated how to use Convolutional Neural Network techniques in medical imaging problems. In this article, the results were obtained through the use of the EfficientNet in the task of classifying 14 different diseases based on chest X-ray images coming from the NIH (National Institutes of Health) ChestX-ray14 dataset. The approach addresses dataset imbalances by introducing a custom split to ensure fair representation. Binary cross entropy loss is utilized to handle the multi-label difficulty. The model architecture comprises an EfficientNet backbone for feature extraction, succeeded by sequential layers including GlobalAveragePooling, Dense, and BatchNormalization. The main contribution of this paper is a proposed solution that outperforms previous state-of-the-art deep learning models average area under the receiver operating characteristic curve—AUC-ROC (score: 84.28%). The usage of the transfer-learning technique and traditional deep learning engineering techniques was shown to enable us to obtain such results on consumer-class GPUs (graphics processing units).

## 1. Introduction

Radiology plays an essential role in diagnosing and monitoring a range of diseases. The demand for radiological services is increasing. A lack of proper equipment or a lack of service causes severe consequences in the treatment process, by introducing additional risks of obtaining a positive result in the treatment of many diseases, in particular neoplastic diseases. The WHO estimates that half of the world has no access to radiological services [1]. “Only 41% of first-level hospitals studied in Nigeria, and 63% of hospitals studied in Botswana had a radiograph machine” [2]. The WHO points to the need to implement innovation to improve this state of affairs. The implementation of low-cost medical imaging technological solutions is needed to make better clinical decisions available to more people around the world. It is important to plan solutions enabling the remote interpretation of medical images, which will compensate for the shortage of radiologists and reduce the costs of operating and maintaining equipment. Radiologists’ associations are making progress in providing technology to interpret radiological images remotely [3].

From October 2017 to November 2018, 42.8 million X-ray examinations were conducted in England. During this period, X-rays accounted for 52.8% of all examination procedures [4]. Chest radiographs are still considered to be the best diagnostic and screening method for some diseases such as pneumonia, cardiomegaly, and pulmonary fibrosis [5,6,7]. Modern deep learning models have the potential to achieve similar or even better effectiveness in assessing chest radiographs than radiologists [8,9]. They can also improve the diagnostic efficiency of radiologists, which directly increases the percentage of correctly diagnosed cases, especially in more complex incidents [10].

Deep learning can be considered a promising technology for radiology since the work of radiologists mainly concerns image interpretation. Deep learning methods, and especially CNNs, excel at such tasks. In 2012, Krizhevsky et al. [11] proposed AlexNet—a CNN capable of achieving human-like performance in the task of image classification. They proved that computers can achieve over 80% accuracy in the task of complicated object classification, something that has not been achieved ever before. If CNNs can accurately recognize complicated objects such as animals, they might also be capable of accurately recognizing diseases from medical images.

The work presented here is one example showing that it is possible to use machine learning technology to evaluate X-ray pictures using inexpensive computer sets and available computational techniques/software, today as an advisory program, and in the future to replace the radiologist in some basic tasks.

## 2. Related Work

Yao et al. suggested the usage of long short-term memory (LSTMs) in the network architecture to exploit dependencies among pathology labels, and achieved promising results without any pretraining [12]. LSTMs are well-suited to capturing sequential patterns inherent in time-based data, which makes them particularly adept for analyzing medical data with time-related characteristics and for clinical prediction based on electronic health records. They are able to comprehend long-term dependencies and have the capacity to select which information to summarize or omit before moving on to the next subsequence [13]. Another approach introduced by Shen et al. includes the dynamic routing of dense blocks in the network architecture [14], which aims to combine the benefits of dense connections [15] and capsules while reducing the network depth. In their model, the trainability of the routing coefficient is restricted solely to the concluding iteration, leading to a reduction in both training and inference times [14]. This work also introduced Gradient-weighted Class Activation Mapping (GradCAM), instead of plain class activation maps, for visualizing pathology attention maps for chest X-rays. The heatmap produced by their model retains an advantage of interpretability similar to CAM, without sacrificing classification accuracy, achieved through the incorporation of global average pooling [16]. Multitask learning has also proved to be robust in abnormality detection in chest radiograms. In their research, Li et al. introduced a unified diagnosis network that simultaneously classified and localized pathologies. They integrated disease identification and localization into a single underlying prediction model, employing two distinct loss functions. By leveraging pathology label information and limited location annotations, the model outperformed the reference baseline [17]. Rajpurkar et al. created CheXnet, an algorithm based on the DenseNet convolutional network, which achieved state-of-the-art results on the NIH ChestX-ray14 dataset. The results of the algorithm proposed by them are statistically significantly better than those achieved by experts in radiology. In addition, the authors have also suggested an extension of the model, allowing it to enhance its capability to detect up to 14 distinct diseases [18]. In their work, Güendel et al. proposed an approach utilizing location-aware dense networks, which incorporated the spatial information and high-resolution images for abnormality classification in chest X-rays. This integration significantly enhanced classification accuracy, especially when location details were available [19].

## 3. Methodology

### 3.1. Dataset

The NIH ChestX-ray14 dataset was used by us to train and evaluate the model [20]. The dataset consisted of 112,120 X-rays from the front of 32,717 unique patients. Each radiogram had a resolution of 1024 × 1024 px. Additional patient information included gender and age. A standard patient-wise split of the dataset was implemented, which ensured that there was no overlap of patients between the training and test sets. In total, there were 86,524 and 25,596 samples in the training and test sets, respectively. Each sample in the dataset had up to 14 pathology labels: atelectasis, cardiomegaly, effusion, infiltration, mass, nodule, pneumonia, pneumothorax, consolidation, oedema, emphysema, fibrosis, pleural thickening, and hernia.

Several concerns have been observed with the official split technique, in which the training and test datasets had different characteristics. This could be either due to a tremendous inconsistency in the label or the test set having an average of 3 times more photos per patient compared to the training set. Because of that, instead of using an official split, a custom split was used with random seed 2137. The new split was better balanced, had lower divergence, and did not use some images from single patients significantly more often than others.

The provided dataset was not well-balanced (Table 1). Dominant classes such as effusion, infiltration, and atelectasis are much more frequently found than the rare ones, e.g., hernia, which represents only 0.20% of the whole dataset.

### 3.2. Regularization

Regularization is an important part of training deep convolutional networks while avoiding overfitting, which can lower the level of the network’s ability to generalize the samples during the training and as a result can decrease the overall model’s ability to predict correctly. Amongst regularization methods are batch normalization [21] and data augmentation [22]. Data augmentation is an explicit form of regularization, which increases the size of the training dataset by transforming existing samples. This includes random flipping, which in our case flips the input image along the *x*-axis with the probability *p* = 0.3, random rotation, which rotates the image up to 5 degrees clockwise or counterclockwise with probability *p* = 0.1, and random brightness modification by a factor in the range <−0.2, 0.2>.

### 3.3. Loss Function

The solved problem was a multi-label problem. This means it was not possible to apply traditional single-class cost functions such as MSE or categorical cross-entropy. The applied model was optimized with a standard loss function for multiclass problems—binary cross entropy.

### 3.4. Architecture

**Figure 1:**
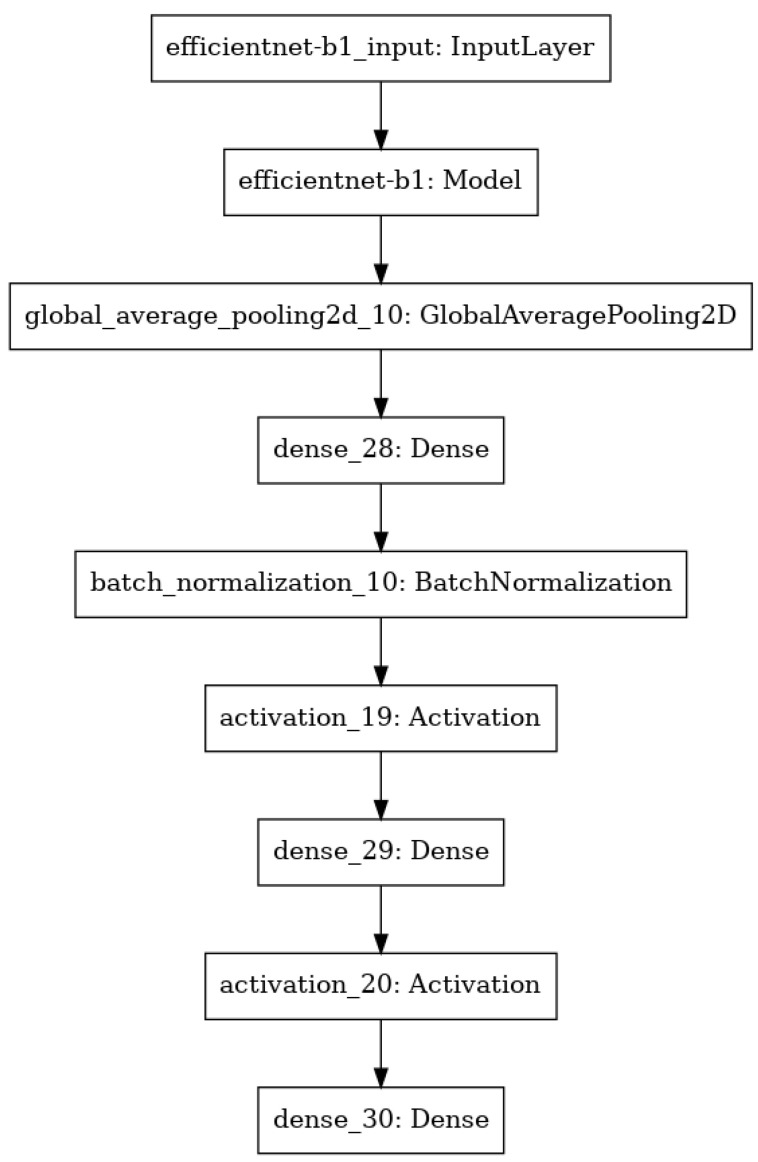
Summary of the model architecture.

In the presented approach, the model was created as a sequence of layers where the output from the previous layer was an input for the next one. The backbone of the model was an EfficientNet [23], which was responsible for processing the input image and extracting the feature map upon which the rest of the network was based. Except for the backbone, the model consisted of the following layers: GlobalAveragePooling, Dense, BatchNormalization and layers with the Rectified Linear Unit (ReLU) activation function. The last layer was the classification layer, which was the Dense layer with 14 nodes, as 14 classes of different chest pathologies were classified. The summary of the model is shown in Figure 1.

### 3.5. Training Procedure

The training procedure consisted of so-called epochs, where one epoch was counted when an entire dataset was passed through the model. After each epoch, the validation process took place, where the average AUC score for all classes was calculated. The callback function checked whether the current state (current values of all parameters) of the model performed better and achieved better average AUC scores for the validation data, or not. In case the model did improve, the state of the model was saved in order to store the best-performing state of the model during the whole training process. The training procedure can take a lot of time, and there is no guarantee that the model will improve after many epochs. Therefore, another callback function was used by us to stop the procedure in case the model did not improve after a certain number of epochs in a row.

## 4. Experiment

### 4.1. Evaluation Metrics

**Figure 2:**
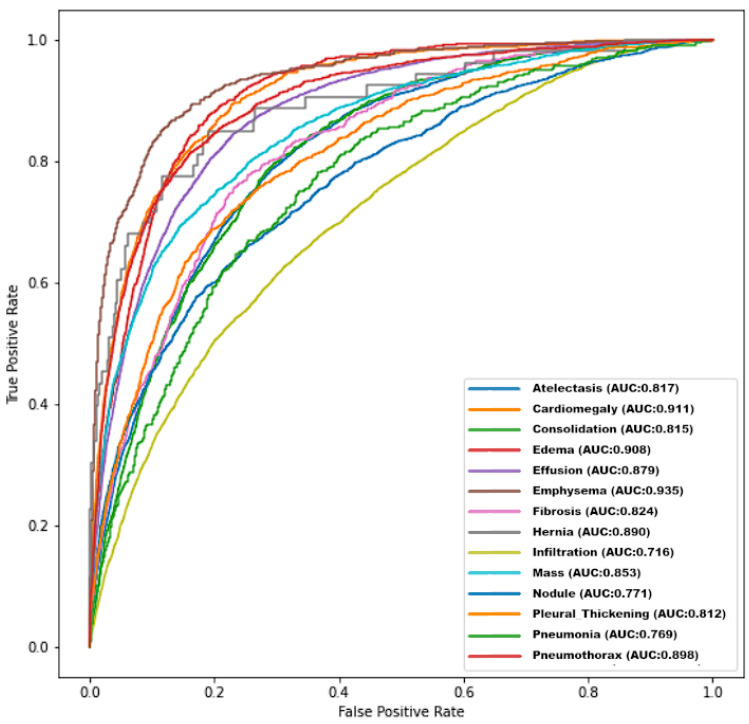
ROC curves for different diseases achieved by the presented model.

In order to evaluate the performance of the model, AUC ROC was computed for each label, comparing the predictions made by the presented network to the expected values in the test set. The ROC curves are shown in Figure 2.

### 4.2. Comparison

In Table 2, the AUC scores achieved by the model were compared with the results of different models described in the literature. In contrast to the other research groups, instead of using an official test-train split the custom split was used, because of official split imperfections. The created model achieved state-of-the-art performance in a pathology classification of the majority of labels and had the best average AUC score. The proposed solution outperformed earlier models in the classification of eight different diseases (atelectasis, cardiomegaly, effusion, mass, pneumothorax, consolidation, oedema, and pleural thickening). The best results were achieved in comparison with other scientific reports in the diagnosis of oedema, pneumothorax, and cardiomegaly. In each of these classifications, 90% efficiency was achieved. Some labels achieved worse results. Worse results were achieved compared to Shen et al. in classification, infiltration (0.751 vs. 0.716), and pneumonia (0.778 vs. 0.769); compared to Yan et al. in the classification of emphysema (0.942 vs. 0.935), fibrosis (0.833 vs. 0.824) and nodule (0.811 vs. 0.771) [24]; and compared to Baltruschat et al. in the classification of hernia (0.937 vs. 0.890) [25]. However, compared to Wang et al., better results were achieved in each disease and mean AUC ROC [26].

Compared to DenseNet-121, an architecture used by Guendel and Rajpurkar, EfficientNet-B1, which was chosen for the feature extraction part of the network, achieved a better performance, despite having fewer parameters (8.4 million vs. 6.7 million). Additionally, as a consequence of the architecture having a significantly smaller number of parameters, the prediction process was faster and the model required less memory.

### 4.3. Interpretation

**Figure 3:**
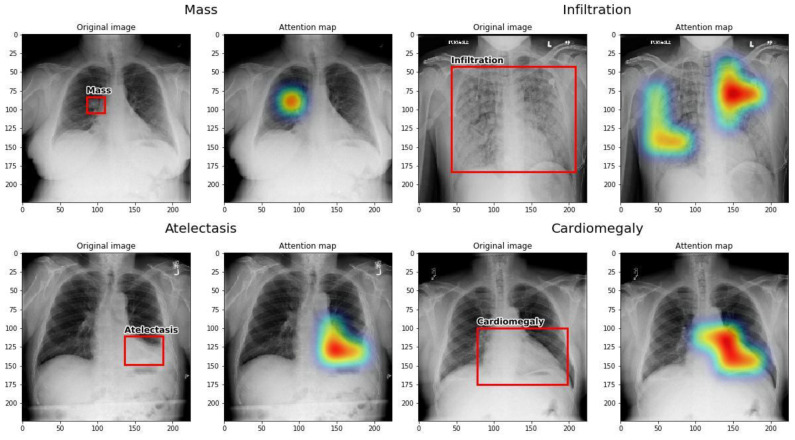
Four examples of localization maps of important regions in an image produced by the network, using Grad-CAM.

The Grad-CAM technique was used to interpret the results of the presented neural network. A map of locations of significant changes for a given label was created, which is shown in Figure 3. Then, the Grad-CAM localization map was compared with pathology bounding boxes from the NIH dataset. The present network was observed by us to correctly locate the pathological changes in the X-ray images from the test set, and it showed the effective location of pathological changes in several cases such as atelectasis, cardiomegaly, effusion, and mass. Both small, localized changes like mass and distributed ones like cardiomegaly were correctly recognized.

## 5. Discussion

An attempt to develop and check the operation of the original diagnostic system based on artificial intelligence was presented in the paper, with a deep learning model capable of diagnosing 14 common thorax abnormalities: atelectasis, cardiomegaly, effusion, infiltration, mass, nodule, pneumonia, pneumothorax, consolidation, oedema, emphysema, fibrosis, pleural thickening, and hernia. This model was a CNN based on the EfficientNet architecture [23], which can predict up to 14 abnormalities based on the input image. To train and evaluate the model, the NIH ChestX-ray14 dataset was used [26], which is a hospital-scale database which contains 112,120 frontal view chest radiographs, each annotated with up to 14 thoracic abnormalities labels. Transfer learning and data augmentation were used to improve the performance of the network [22,27]. Then, the model was evaluated by comparing its AUC ROC values with other published results for that dataset. Finally, the attention was visualized through a map of the network using Grad-CAM to help localize the pathology [28].

### 5.1. Comparison with Existing Literature

Compared to other researchers cited in this article, the highest average score was obtained by this study. Results were also higher in some pathologies, such as atelectasis, cardiomegaly, effusion, mass, pneumothorax, consolidation, oedema and pleural thickening.

Similarly to some previous [24,25] solutions, the proposed model was initialized with ImageNet weights. Adam was chosen as an optimizer. In contrast to other teams, the custom data split with a random seed was used.

It is noteworthy that only Shen et al. proposed to use CNN capsules, and they obtained the best ROC AUC in infiltration and pneumonia [14].

Despite data augmentation and transfer learning, the key factor that enabled the presented model to outperform previous solutions was the usage of EfficientNet as a backbone model. EfficientNet is currently considered to be one of the best existing architectures for image classification problems. There exist eight different versions of EfficientNet, from B0 to B7, each with a different number of layers and a different number of parameters. Due to the GPU memory limitations, EfficientNet B1 version was used.

In the opinion of the authors, the combination of transfer learning and standard practices, such as including transfer learning, data augmentation, and additional callback functions, enabled the model to achieve state-of-the-art results.

A comparison table highlighting the strengths and weaknesses of the proposed and previous methods has been included below (Table 3).

### 5.2. Restrictions

Several problems related to the dataset itself arose after the analysis of these medical data. Due to the presence of numerous disease entities, and thus the adherence of several labels to one X-ray image, irregularities may occur which impact the training process of the model. Furthermore, it is not understood what the pneumonia label and consolidation mean because it is not possible to visually distinguish these two pathologies, so it is not clear how they affected the training process.

In addition, some X-ray images were rotated 90 or 180 degrees. Some also included foreign bodies in the form of jewelry or metal elements in clothing, which introduced additional noise during the training process. Due to the difficulty of excluding all artifacts in dataset radiographs, they were not included in the exclusion criterion in the study, thus negatively affecting the training of the artificial intelligence under study. However, this allowed for a better representation of the daily work of a radiologist, in which such artifacts can sometimes be found.

In contrast to Wang et al. [20] and Baltruschat et al. [25], only two GPUs (Nvidia 1080 Ti) were available, which was a highly limiting factor. Due to the GPU memory limitations, the EfficentNet B1 version was used. The usage of a higher version, like EfficientNet B7, may in the future contribute to the improvement of the results.

### 5.3. Comment

Compared to other deep learning models, promising results were achieved. Probably, in the future, by combining the knowledge of domain experts with existing deep learning techniques, it will be possible to obtain more accurate, faster, and cheaper results from an analysis of medical imaging. Models such as this one could improve the healthcare system in the future by helping radiologists create radiology reports more efficiently, analyze patient disease correlations, and search for common diseases. However, before enabling artificial intelligence to autonomously diagnose a patient, it is important to make sure it is in line with ground truth, which is fundamentally rooted from the human perspective.

### 5.4. Conclusions

The results were compared with the achievements of other teams available in the literature and it was shown that it is possible to obtain state-of-the-art results for complicated medical diagnosis problems by applying existing deep learning architecture such as the EfficientNet architecture, transfer learning technique, data augmentation or extended callback functions. The ImageNet weights were used as a starting point for training, and binary cross-entropy was used as a loss function to strengthen underrepresented classes. Early stopping and model checkpoint callback functions were also used to further improve the training procedure. The performance of the model presented by the researchers holds significance in the context of the non-standard data partitioning employed in the study and potentially reveals limitations of the official division. The utilization of random data splitting based on a seed allowed for an even distribution of classes, which is a favorable factor for the training process and for reliable model validation. Discrepancies in the achievements of the models described in the literature were also noted. These differences suggest that alongside appropriate data partitioning, the selection of training methods and network architecture remains crucial. Among the cited literature models, the most effective approach was demonstrated by Yan et al.’s [24] model. One of the significant characteristics of the mentioned network is the extension of the routing mechanism through a convolutional layer. Some cited models exhibited higher effectiveness in specific classes, regardless of the averaged performance. This implies that certain machine learning methods can be particularly effective in the mentioned tasks. Defining the network features leading to such results and refining general models based on this information remains a pertinent issue. The presented results were obtained by using a consumer-class GPU (Nvidia GTX 1080 Ti). It is very promising that just by applying such methods, the model was able to outperform all previous works and achieve state-of-the-art performance in the task of X-ray chest disease recognition. The team of authors is aware that the system should be further trained and validated on different datasets. Therefore, it is planned to make the source code available to people interested in cooperating in improving the application.

## Figures and Tables

**Table 1:** Dataset imbalance statistics.

Pathology Label	Percentage	Count
No Finding	53.84%	60,361
Atelectasis	10.31%	11,559
Cardiomegaly	2.48%	2776
Effusion	11.88%	13,317
Infiltration	17.74%	19,894
Mass	5.16%	5782
Nodule	5.65%	6331
Pneumonia	1.28%	1431
Pneumothorax	4.73%	5302
Consolidation	4.16%	4667
Edema	2.05%	2303
Emphysema	2.24%	2516
Fibrosis	1.50%	1686
Pleural Thickening	3.02%	3385
Hernia	0.20%	227

**Table 2:** Model comparison.

Pathology Label	Yao et al. [12]	Wang et al. [26]	Shen et al. [14]	Guendel et al. [19]	Yan et al. [24]	Baltruschat et al. [25]	Ours
Official split	Yes	Yes	Yes	Yes	Yes	Yes	No
Atelectasis	0.733	0.700	0.766	0.767	0.792	0.763	0.817
Cardiomegaly	0.856	0.810	0.801	0.883	0.881	0.875	0.911
Effusion	0.806	0.759	0.797	0.828	0.842	0.822	0.879
Infiltration	0.673	0.661	0.751	0.709	0.710	0.694	0.716
Mass	0.777	0.693	0.760	0.821	0.847	0.820	0.853
Nodule	0.724	0.669	0.741	0.758	0.811	0.747	0.771
Pneumonia	0.684	0.658	0.778	0.731	0.740	0.714	0.769
Pneumothorax	0.805	0.799	0.800	0.846	0.876	0.840	0.898
Consolidation	0.711	0.703	0.787	0.745	0.760	0.749	0.815
Edema	0.806	0.805	0.820	0.835	0.848	0.846	0.908
Emphysema	0.842	0.833	0.773	0.895	0.942	0.895	0.935
Fibrosis	0.743	0.786	0.786	0.818	0.833	0.816	0.824
Pleural Thickening	0.724	0.684	0.759	0.761	0.808	0.763	0.812
Hernia	0.775	0.872	0.748	0.896	0.934	0.937	0.890
Average	0.761	0.745	0.775	0.807	0.830	0.727	0.843

**Table 3:** A comparison of the strengths and weaknesses of the suggested and previous methods.

Author	Strengths	Weaknesses
Kufel et al.	Highest average score among similar research; use of the custom data split with a random seed; EfficientNet used as a backbone model.	Use of EfficentNet B1 version due to GPU limitations (two Nvidia 1080 Ti).
Yao et al. [12]	Use of CNN capsules; the best ROC AUC in infiltration and pneumonia achieved.	Challenges in effective training with small datasets, and limited applicability compared to traditional neural network architectures.
Shen et al. [14]	Better results in classification of infiltration and pneumonia (compared to Kufel et al.).	Method includes its complexity in terms of computational requirements due to the multiple layers, as well as the potential challenges in efficiently updating coupling coefficients and performing dynamic routing in the context of 1 × 1 convolutional layers, which may lead to computational exhaustiveness and limitations in feasibility for practical implementations.
Yan et al. [24]	Better results in classification of emphysema, fibrosis, and nodule (compared to Kufel et al.).	Approach involves a reliance on comparisons with other methods, where methodological variations such as data splitting setups and additional disease information can impact the fairness and interpretability of the performance assessment. Moreover, while the method demonstrates improvements in overall performance and addresses challenges like spatial squeezing and lesion size variation, it might not fully address nuanced differences in disease presentation and diagnostic intricacies present in real-world scenarios.
Güendel et al. [19]	Use of location-aware DNN, combining spatial information and high-resolution images.	Method includes a potential susceptibility to overfitting due to the use of a complex architecture and high-resolution images, especially given the imbalance in the dataset. Additionally, the approach might face challenges in accurately representing complex spatial information and disease locations, particularly when dealing with multiple and diffuse diseases for which precise position information is lacking.
Wang et al. [20]	Larger GPU was used for the training process.	Worse results achieved in each disease and worse mean AUC ROC (compared to Kufel et al.).
Baltruschat et al. [25]	Larger GPU was used in the training process, better results in classification of hernia (compared to Kufel et al.).	The weaknesses associated with this method include potential challenges arising from the use of transfer learning and fine-tuning. While transferring knowledge from a different domain might provide a head start, it could also introduce biases or assumptions from the source domain that do not hold true in the medical context, potentially leading to suboptimal performance or misinterpretations. Furthermore, adapting complex architectures like ResNet-50 for medical imaging with limited datasets may increase the risk of overfitting, as the model might be highly sensitive to variations in the small dataset. Additionally, although incorporating patient-specific information (age, gender, view position) may seem advantageous, it could also introduce noise or irrelevant factors that may not consistently aid in improving classification performance across various scenarios.

## Data Availability

The dataset is open access. Other data files are available to interested parties from the authors of this article.
